# SOAX: A software for quantification of 3D biopolymer networks

**DOI:** 10.1038/srep09081

**Published:** 2015-03-13

**Authors:** Ting Xu, Dimitrios Vavylonis, Feng-Ching Tsai, Gijsje H. Koenderink, Wei Nie, Eddy Yusuf, I-Ju Lee, Jian-Qiu Wu, Xiaolei Huang

**Affiliations:** 1 Department of Computer Science and Engineering, Lehigh University, Bethlehem, Pennsylvania, USA; 2 Department of Physics, Lehigh University, Bethlehem, Pennsylvania, USA; 3 FOM Institute AMOLF, Systems Biophysics department, Amsterdam, The Netherlands; 4 Surya College of Education, SURE Center, Tangerang, Indonesia; 5 IPH Schools, Surabaya, Indonesia; 6 Department of Molecular Genetics, The Ohio State University, Columbus, Ohio, USA

## Abstract

Filamentous biopolymer networks in cells and tissues are routinely imaged by confocal microscopy. Image analysis methods enable quantitative study of the properties of these curvilinear networks. However, software tools to quantify the geometry and topology of these often dense 3D networks and to localize network junctions are scarce. To fill this gap, we developed a new software tool called “SOAX”, which can accurately extract the centerlines of 3D biopolymer networks and identify network junctions using Stretching Open Active Contours (SOACs). It provides an open-source, user-friendly platform for network centerline extraction, 2D/3D visualization, manual editing and quantitative analysis. We propose a method to quantify the performance of SOAX, which helps determine the optimal extraction parameter values. We quantify several different types of biopolymer networks to demonstrate SOAX's potential to help answer key questions in cell biology and biophysics from a quantitative viewpoint.

Network structures made of filamentous biopolymers are ubiquitous among biological systems. Biophysicists and cell biologists routinely use static and time-lapse confocal fluorescence microscopy to image intracellular networks of actin filaments^1^,^2^ and microtubules^3^,^4^ as well as extracellular polymers such as fibrin^5^,^6^, both in vitro and in live cells. To gain insight in the structural, dynamical, and mechanical properties of these networks and to understand the mechanisms of their formation requires image analysis methods for automated quantification of massive image datasets. However, user-friendly, flexible, and transparent^7^ software tools to reliably quantify the geometry and topology of these (often dense) networks and to localize network junctions in 3D are scarce.

Previous methods for extracting biopolymer network structures include morphological thinning of a binary segmentation^8^,^9^,^10^,^11^ or a computed tubularity map^12^,^13^, Radon transform^14^ and template matching^15^,^16^. However, most of these methods extract disconnected points (i.e. pixels) on centerlines without inferring network topology and they have not been implemented as part of a software platform. One available software tool is “Network Extractor” (http://cismm.cs.unc.edu/), which finds one-pixel wide 3D network centerlines by thresholding and thinning a tubularity map. Thresholding results, however, can suffer from inhomogeneous signal-to-noise ratio (SNR). Other software for extracting curvilinear network structure are designed for neuronal structures^17^,^18^,^19^,^20^. Vaa3D-Neuron^19^ (http://www.vaa3d.org/) is a semi-automatic neuron reconstruction and quantification tool which requires the user to pinpoint the end points of a neuronal tree so that a minimal path algorithm can reconstruct the structure. The Farsight Toolkit (http://farsight-toolkit.org/) also contains 3D neuron tracing and reconstruction software command-line modules^21^,^22^.

To fill this gap in available software, here we provide an open source program, SOAX, designed to extract the centerlines and junctions of biopolymer networks such as those of actin filaments, microtubules, and fibrin, in the presence of image noise and unrelated structures such as those that appear in images of live cells. SOAX provides quantification and visualization functions in an easy-to-use user interface.

The underlying method of SOAX is the multiple Stretching Open Active Contours (SOACs) method that was proposed to extract the 3D meshwork of actin filaments imaged by confocal microscopy^23^. Here we implement this method in SOAX and apply it generally to different types of biopolymer networks. While the SOAX method is robust against noise, its parameters need to be adjusted depending on the type of biopolymer and the image SNR. Parameters for actin filaments were previously chosen empirically^23^. Here we provide a new method to evaluate the accuracy of the network extraction results and find a small set of candidate optimal solutions for the user to choose from, without relying on prior knowledge of ground truth. The selected optimal extraction result can be subsequently used for quantitative analysis of biopolymer filaments, such as their spatial distribution, orientation and curvature. Time lapse movies can be conveniently analyzed by reusing the selected parameters from one image for other images drawn from the same dataset. We demonstrate SOAX's potential to help provide quantitative results to answer key questions in cell biology and biophysics from a quantitative viewpoint.

## Results

### Description of SOAX software

**Figure 1:**
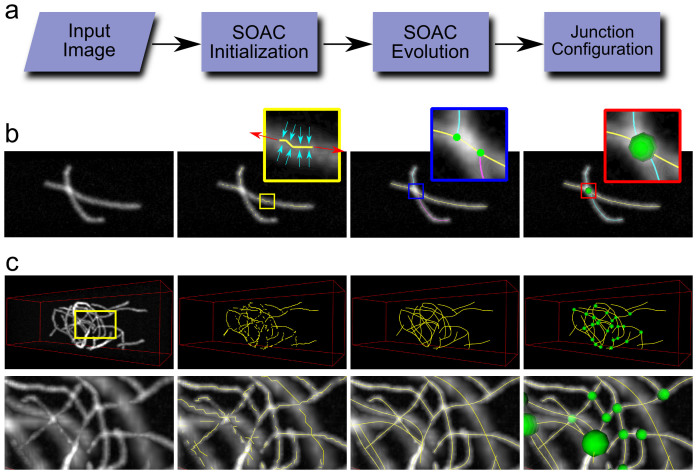
Overview of SOAX for network centerline, topology and junction extraction. (a) Given an input image, multiple Stretching Open Active Contours (SOACs) are automatically initialized. The SOACs evolve by moving, stretching, merging and forming junctions with one another. The final network topology is configured by cutting and joining the contours such that SOACs do not end or bend sharply at junctions. (b) Result after each stage on an image of two crossing filaments in a simulated image. The yellow zoomed-in window shows the forces exerted on a SOAC during its evolution. Stretching forces (red) elongate it while image forces (cyan) keep it on the centerline of the filaments. Blue window shows two T-junctions (green dots) formed after sequential SOAC evolution. Red window shows reconfigured SOACs and localized filament junction (green sphere) after clustering nearby T-junctions. (c) Upper row: extraction process on a 3D synthetic filament meshwork. Lower row: zoomed-in view of the yellow window at upper row. First column: input image. Second column: initialized SOACs. Third column: evolved and merged SOACs. Fourth column: reconfigured SOACs with junctions (green spheres).

SOAX extracts network structures in three stages: SOAC initialization, SOAC evolution, and junction configuration (Fig. 1a, Supplementary Note 1, Supplementary Movie 1)^23^. A SOAC is a parametric curve that “evolves”: it is attracted towards the centerline of a filament, stretches by elongation, and stops stretching when its end reaches a filament tip. Figure 1b and 1c show examples of the extraction process for synthetic images.

In the initialization stage (second column in Fig. 1), multiple short SOACs are automatically placed along intensity ridges of the image, which correspond to centerlines of filaments in 3D or 2D, depending on the dimensionality of the image. A ridge threshold parameter (τ) specifies the minimal intensity steepness for a ridge to host an initial SOAC. These initial SOACs can be fragmented, jagged, and redundant.

In the evolution stage (third column in Fig. 1), the SOACs evolve one after another and stretch according to the local intensity contrast, weighted by a global parameter (*k*_str_) that controls how easily SOACs elongate overall. The yellow panel in Figure 1b shows the stretching forces (red) applied at the SOAC tips as well as the image forces (cyan) that keep it on the centerlines. SOACs stop stretching at a filament end or when their tip collides with the body of another SOAC to form a T-junction (blue box in Fig. 1b). Checks are performed to eliminate SOAC overlap. After evolution, they smoothly lie on the centerlines of filaments and connect with one another at T-junctions.

The final stage (fourth column in Fig. 1) is clustering nearby T-junctions into a single junction followed by configuring the local connectivity of SOACs by cutting and splicing them such that they do not end or bend sharply at junctions (red box in Fig. 1b). This stage results in a set of junction points and re-configured SOACs that better represent the topology of physical filaments.

SOAX provides 3D volume rendering and slicing planes for exploring image data and visually checking the result against the image. A local visualization function reduces clutter when viewing results in 3D. It also supports viewing the color-coded orientation difference of resultant SOACs. Since cell images may contain structures other than networks, we implemented manual editing functionalities to allow users to improve the automated results. Users can cut, extend and modify the body of each SOAC and delete junctions. For more details on these operations, please see http://www.cse.lehigh.edu/~idealab/soax/.

### Evaluating extraction results and choice of optimal parameters

Our network extraction method includes several parameters such as the active contour bending and stretching stiffness, distance threshold for junction formation, SOAC overlap distance, and size of region for local background calculation^23^. While these parameters can be estimated for a set of images (using known values of filament persistence length, pixel size and density of the network being imaged), network extraction depends crucially on the SNR of the image and the appropriate choice of two parameters mentioned above: ridge threshold τ and stretch factor *k*_str_. Large values of the ridge threshold τ will result in SOAC initialization only at very bright filaments, while low values may initialize SOACs on background noise. Too large a stretch factor *k*_str_ will elongate SOACs beyond the tips of filaments, while too small values may cause a SOAC to prematurely stop extending due to intensity fluctuations along the filament and the local background. Reliable network extraction requires a procedure to estimate appropriate values for τ and *k*_str_ for images of varying SNR. Existing methods for real-time segmentation evaluation usually rely on the availability of ground truth^24^,^25^ or a predicted one generated by supervised machine learning^26^, which in turn relies on ground truth data. Most of the unsupervised evaluation approaches in the literature are based on metrics defined on image regions, thus are not directly applicable to the evaluation of contours^27^. Here we developed an evaluation method based on an optimization function to help the user select the best extraction result from a set of candidates.

**Figure 2:**
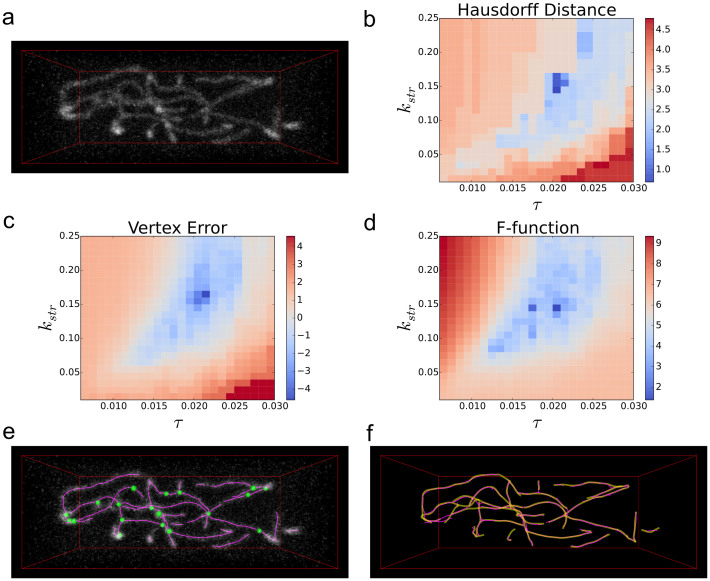
Parameter optimization using the F-function. (a) Synthetic test image with mean local SNR = 3.34. (b–c) Hausdorff distance and vertex error (log-scale plots: ln(*x − x*_min_ + ε), where *x* is the data value; *x*_min_ is the minimum value of all data; ε is a constant offset) computed between ground truth and results extracted using various values of the ridge threshold τ (normalized intensity change per pixel) and stretch factor *k*_str_ (pixels per time step). (d) The proposed F-function (log-scale) computed using *t* = 2.2, *c* = 2.0. The optimal parameters are τ = 0.02, *k*_str_ = 0.14 acquired by minimizing the F-function. The optimal result has Hausdorff distance 7.15 pixels and vertex error 1.08 pixels compared to ground truth. (e) Optimal extraction result overlaid with original image in (a). (f) Optimal extraction result (magenta) overlaid with ground truth (translucent yellow).

We started by generating synthetic network images with simulated shot noise (Fig. 2a) and applied SOAX with varying ridge thresholds and stretch factors. Knowing the ground truth network for these images allows us to evaluate the accuracy of each extraction result using the Hausdorff distance (Fig. 2b) and vertex error (Fig. 2c). The Hausdorff distance is the largest distance between any point on the SOAX-extracted network and its closest point on the ground truth network and vice versa; the vertex error is the average distance between points on the extracted network and their closest points on the ground truth network and vice versa. Figure 2b,c show an optimal range for τ and *k*_str_ (dark blue regions).

Evaluating the optimality of parameters based on the Hausdorff distance or vertex error relies on knowledge of the ground truth, which is not available for experimental images. Thus, we searched for a distance measure that mimics the heat maps in Figure 2b,c but does not require ground truth. We propose a new measure we call the “F-function” that evaluates an extraction result using only the image and the result. We define *F* = −*L*_total_ + *cL_<t_*, where *L*_total_ is the total length of SOACs in the extraction result, *L_<t_* is the length of SOAC segments in regions of the image with local SNR below a threshold *t*, and *c* is a factor larger than unity controlling how much low-SNR SOACs are penalized (see Online Methods and Supplementary Note 2). Minimizing the F-function favors extraction results that are as complete as possible (large *L*_total_) but penalizes the portions with low local SNR. When *t* and *c* are chosen properly, the F-function can have a similar dependence on τ and *k*_str_ as the Hausdorff distance and vertex error and have similar optimal region representing good extraction results as the other two metrics (Fig. 2d). The optimal extraction result selected from the optimal parameters in Figure 2d corresponds to the image very well (Fig. 2e) and is very close to the ground truth, with a Hausdorff distance of 7.15 pixels and vertex error of 1.08 pixels (Fig. 2f).

Evaluation methods based on the ROC curve^28^ or F-measure^29^ used in pattern recognition and information retrieval can measure the fraction of network centerlines extracted that are relevant (precision) and the fraction of relevant network centerlines that are extracted (recall). The ROC curve is a plot of true positive rate (recall) against false positive rate while the F-measure is the harmonic mean of precision and recall and can be seen as a compromise between them. However, we did not found them to be appropriate since the true negatives, pixels that are not network centerlines, in the image background dominate these scores. To solve this difficulty, the proposed F-function focuses on the extracted centerlines, and thus only considers true and false positives.

In our evaluation and optimization method we introduced two additional parameters, *t* and *c*, on which the values of suggested optimal parameters depend. Through analysis of synthetic images, we found that valid *t* and *c* can be restricted to a fixed small range {(*t, c*) | 1 < *t* < 5, 1 < *c* < 5, 3 < *t* + *c* < 6} regardless of the input image SNR (Supplementary Note 2). An additional, but narrower, search for optimal *t, c* within this restricted range needs to be performed. For images with local SNR > 5 the largest values of *t, c* in that range work well, but user input is typically required for smaller SNR since (i) the optimal extraction is more sensitive to the choice of *t, c* and (ii) while SOAX allows measuring the local SNR by clicking on initialized or converged SOACs, evaluation of the local SNR can vary depending on the local microenvironment and on the width of the Point Spread Function (PSF). The extraction procedure implemented in SOAX is to (i) obtain a set of different extraction results by varying τ and *k*_str_, and (ii) manually select a good one from a smaller set of candidate optimal extraction results, which are generated by minimizing the F-functions with varying values of *t* and *c* within a small range.

### Spatial distribution of actin filaments in emulsion droplets

**Figure 3:**
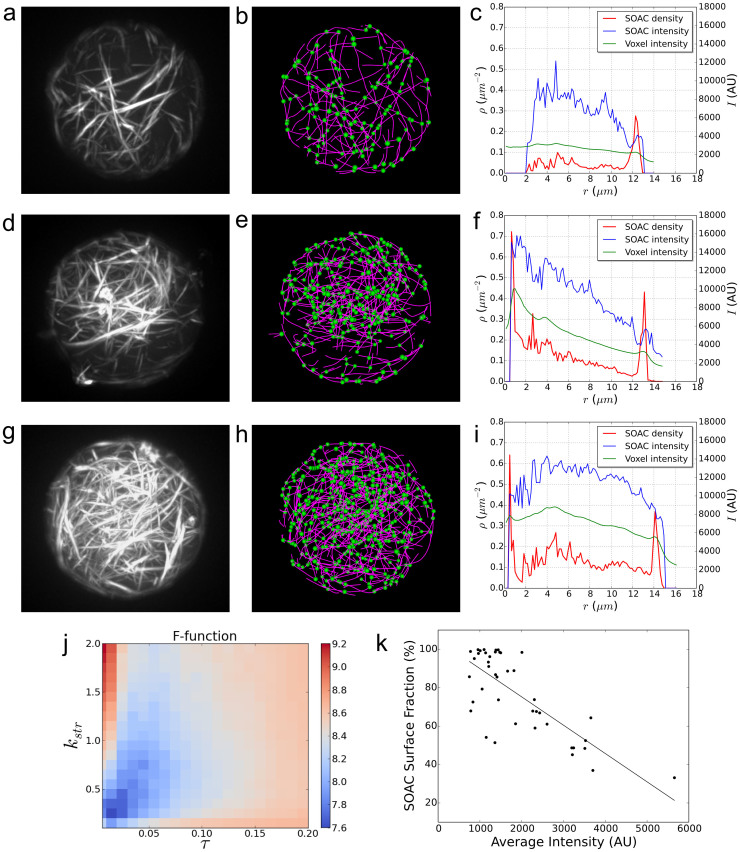
Analysis of concentration and bundle thickness of actin filaments polymerized in an emulsion droplet in the presence of fascin cross-linkers (droplet radius *r* ≈ 13.5 μm) imaged by confocal microscopy. (a,d,g) Volume rendered image shows network of actin filament bundles with different concentration. (b,e,h) Extraction results using the optimal parameters. Centerlines of actin bundles outside of the main droplet were manually deleted using SOAX. (c,f,i) SOAC point density *ρ* (red), average intensity at SOAC points (blue) and average intensity at image voxels (green), as function of distance from droplet center. Graphs show an enhanced concentration of thin bundles parallel to the droplet boundary. The distribution of thicker bundles in the interior differs, with a sparse network in (a), a high concentration near the center in (b), and a distributed network in (g). (j) F-function (*t* = 1.8, *c* = 2.0) shows the optimal τ = 0.01, *k*_str_ = 0.2 for droplet in (d). (k) Scatter plot of fractions of surface SOAC points (distance to droplet center ≥0.9 *r*) against average droplet intensity. Linear least squares fitting shows an inverse correlation with slope −0.015, and R^2^ = 0.58.

**Figure 4:**
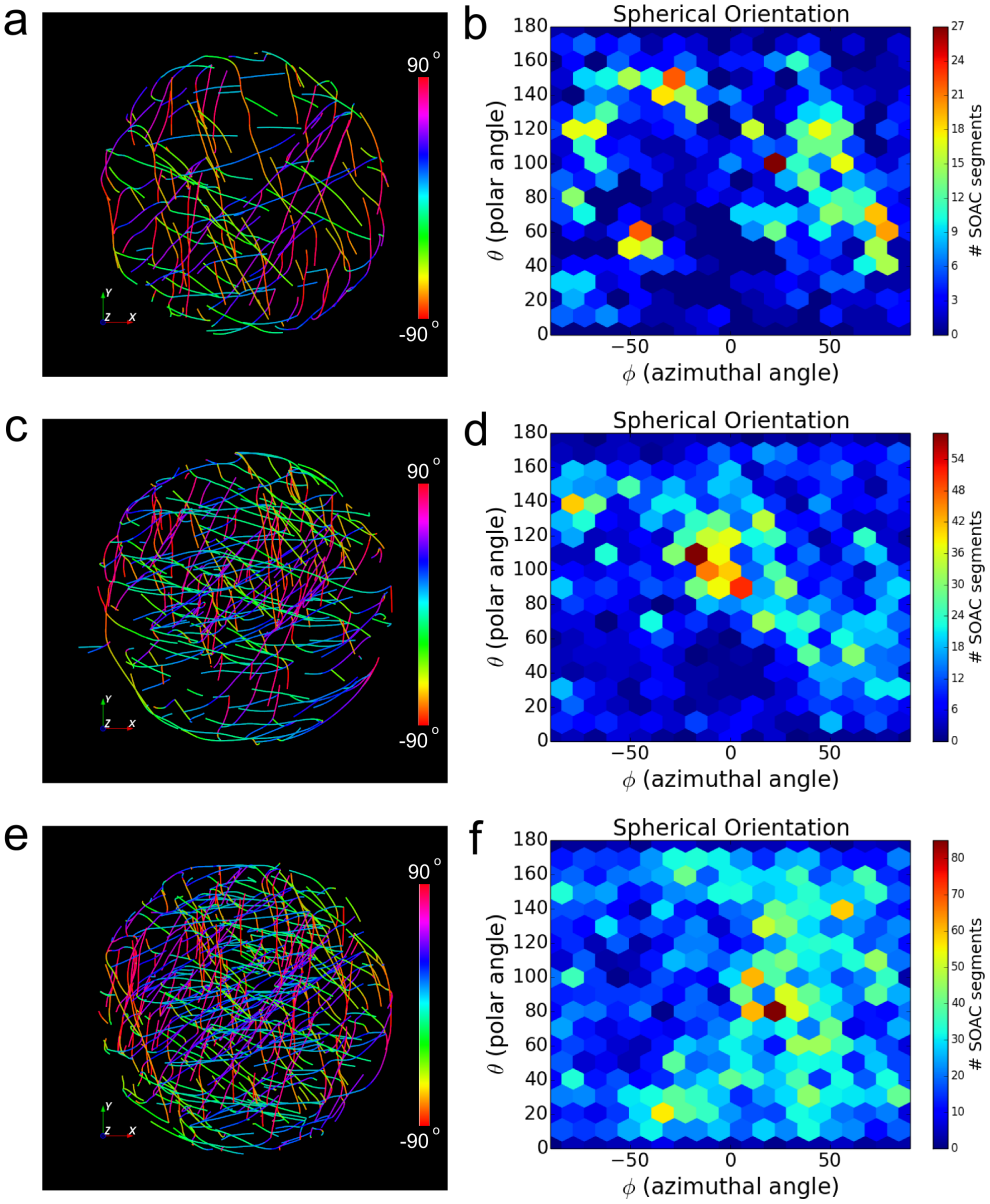
Analysis of actin bundle orientation of droplet images in Figure 3. (a,c,e) Color-coded SOACs based on azimuthal angle *φ* (top view). (b,d,f) 2D histogram of SOAC orientation vs azimuthal and polar angles. The count shows the number of SOAC segments between consecutive SOAC points with a particular *φ* and *θ* within 0.9 *r* of droplet center. The sparse network in (a) does not show a preferred orientation. Enhanced alignment is observed at *φ* ≈ −10° and *θ* ≈ 110° for droplet in (c). The droplet in (e) has more bundles around *φ* ≈ 25°.

To demonstrate the use of SOAX in the quantitative analysis of biopolymer organization in vitro, we polymerized fluorescently-labeled actin in emulsion droplets in the presence of the cross-linking protein fascin (Fig. 3a, 3d, 3g). Actin filament bundles as well as microtubules can reach lengths of order 10 μm, comparable to the size of plant, animal and yeast cells, implying interactions with confining surfaces and organelles. Experiments such as those in Figure 3 can provide a systematic understanding of how a confining geometry restricts long cytoskeletal filaments and directs their orientation^30^. Several theories predict the distribution of semiflexible polymers in confined spaces, their density-dependent nematic ordering and alignment along the confining surface^31^,^32^. However analysis of experiments to quantify distributions of filament orientation, curvature and density is limited by the lack of image analysis tools. Figures 3 and 4 show how SOAX can be used to overcome these limitations.

We first selected one of 42 droplet images prepared under the same experimental conditions that have the same actin concentration for droplet encapsulation (but varying encapsulation efficiency) and a nearly uniform droplet radius *r*. We extracted the actin bundles using 420 combinations of ridge threshold τ and stretch factor *k*_str_ distributed in the range 0.006 < τ < 2 and 0.1 < *k*_str_ < 2. These upper and lower limits were chosen manually to correspond to clear under- or over-detection. We then manually select a good extraction result from the set of 19 optimal candidates generated by minimizing F-functions with various values of *t* and *c* as described in the previous section. The F-function corresponding to the manually selected result is shown in Figure 3j. A finer scan of τ and *k*_str_ around the selected values did not modify extraction results in this example. The same optimal τ and *k*_str_ were used to extract the remaining 41 droplet images.

The optimal extraction result selected for quantitative analysis for three droplets are shown in Figure 3b, 3e, 3h and Supplementary Movie 2. To analyze the distribution of actin filaments inside the droplet, we use the resultant SOACs to compute the filament density distribution and filament intensity distribution along the radial direction (Fig. 3c, 3f, 3i). These two measurements reflect the concentration and thickness distribution of actin filament bundles inside the droplet. The three examples in Figure 3 show different patterns of actin filament bundle distribution. Figure 3c shows that most bundles of the droplet in Fig. 3a are arranged along the droplet boundary at *r* ≈ 13 μm, with a few thick bundles at *r* ≈ 4.8 μm from the droplet center (bundle thickness is proportional to the intensity at the SOAC points). In the droplet of Fig. 3d, both the density and the bundle thickness of the interior bundle network decrease with distance from the droplet center (Fig. 3f). The droplet in Fig. 3g is similar to Fig. 3d but exhibits a higher overall density and thickness in its interior bundle network, with a concentration peak at *r* ≈ 4.8 μm (Fig. 3i). For all three droplets, an enhanced concentration of single filaments or thin bundles is seen aligning with the droplet boundary. To further investigate the origin of boundary alignment, Fig. 3k shows an inverse linear correlation between average total actin signal intensity and the fraction of actin filaments or bundles aligning with the droplet boundary.

To quantitatively analyze the orientation of actin filaments inside the droplet, we measured the orientation of each SOAC segment along the azimuthal angle *φ* and polar angle *θ*, defined in a spherical coordinate system (Fig. 4a, 4c, 4e). Filament/bundle alignment corresponds to clustering in *φ* and *θ* values. The orientation distribution of actin bundles in the droplet interior (distance to droplet center <0.9 *r*) is shown by a 2D histogram in *φ* and *θ* (Fig. 4b, 4d, 4f). The middle droplet exhibits a stronger alignment compared to the other droplets, with 15% of all SOAC segments along −25° < *φ* <15° and 80° < *θ* < 120° (Fig. 4d), while the orientation histograms of the other two droplets show multiple local maxima (Fig. 4b, 4f).

The methods to calculate the density and orientation distributions in Figures 3 and 4 are implemented in the software. An additional analysis implemented in SOAX is the radial SOAC orientation distribution, defined as the angle between the tangent of a SOAC segment and the radial direction (Supplementary Figure 1). Thus SOAX can be used in future studies of in vitro biopolymer networks in confined spaces, or under imposed deformation, to quantify filament distribution, topology, orientation and how these parameters relate to mechanical properties of the filaments.

### Microtubule orientation and curvature in adhered cells

**Figure 5:**
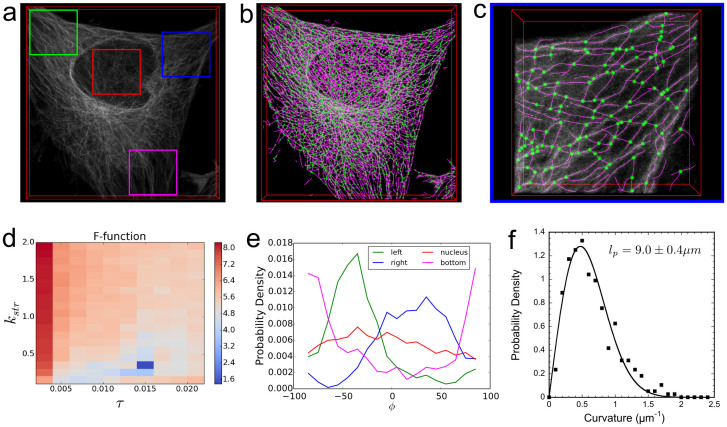
Analysis of microtubules in an adhered HeLa cell stably expressing β-tubulin-GFP imaged by confocal microscopy. (a) Volume rendered image (49.6 × 49.6 × 5.5 μm). (b) Extracted SOACs (magenta) and junctions (green). (c) Extraction result on the blue window in (a). (d) F-function (*t* = 2, *c* = 1.3) suggests optimal *τ* = 0.014, *k*_str_ = 0.3. (e) Histogram of SOAC orientation vs azimuthal angle for microtubules in the 4 local windows of (a) shows alignment along *φ* ≈ 45° (blue), *φ* ≈ −45° (green), *φ* ≈ 90° (magenta) and no significant alignment for the nucleus region (red). The p-values (Mann-Whitney U test) of SOAC orientation *φ* distribution between each pair of local windows are less than 0.01. (f) Curvature distribution of SOAC segments in (c) evaluated over segments of 8 pixels in length, excluding junctions. Fit to a 3D worm-like chain model gives an effective persistence length *l*_p_ = 9.0 ± 0.4 μm.

Analysis of biopolymer networks in live cells is challenging when manual methods to extract every filament are too time consuming and when the SNR is too low for automated methods such as thresholding and thinning to work reliably. An example of this is the microtubule network in an adhered cell shown in Figure 5a. Examining how the entire network is distributed and how it is reorganized over time can provide insight in the mechanisms that cells use to change shape and move or divide.

The SOAX program can extract the microtubules inside the entire cell (Fig. 5b). To better show the effectiveness of SOAX, Figure 5c shows an enlarged region corresponding to the blue window in Figure 5a, in which most microtubules are oriented along the northeast direction. As before, we picked a good extraction result from a set of 27 optimal candidates selected from 220 combinations of ridge threshold τ and stretch factor *k*_str_ (distributed in the range 0.002 < τ < 0.022 and 0.1 < *k*_str_ < 2, similar to Fig. 3) using the F-function (Fig. 5d).

As a quantitative validation that most microtubules in Figure 5c point northeast, on average, we computed the orientation histogram of azimuthal angle *φ*. (We ignore the polar angle here because the majority of microtubules lie along the *x-y* plane, since this region is a very thin and flat part of the cell.) As shown by the blue curve in Figure 5e, the distribution peaks at *φ* ≈ 45°. To illustrate the ability of SOAX to differentiate between different regions of the cell, we also computed the histogram of *φ* for other parts of the original image in Figure 5a (corresponding box and curve colors). As shown in Figure 5e, microtubule orientation in the green window peaks at *φ* ≈ −45° and in the magenta window at *φ* ≈ 90° while there is no significant orientation preference in the red window at the cell nucleus.

The distribution of curvature (defined as the magnitude of the rate of change of the unit tangent vector with respect to arc length over a distance large enough to be independent of the intrinsic stiffness of the SOAC, see Online Methods) and effective persistence length (showing how fast a microtubule changes direction) are also easy to extract using built-in functions. Figure 5f shows peak in the curvature distribution around 0.5 μm^−1^. A fit to the 3D worm-like chain model^33^ gives an effective persistence length of 9.0 μm, close to the 30 μm prior measurement of single microtubules in cells in 2D^34^. This length is much shorter than the persistence length of purified microtubules in vitro, of order mm, a result of frozen-in fluctuations during microtubule elongation and motor pulling^34^,^35^,^36^.

### Distribution of actin cables in fission yeast

**Figure 6:**
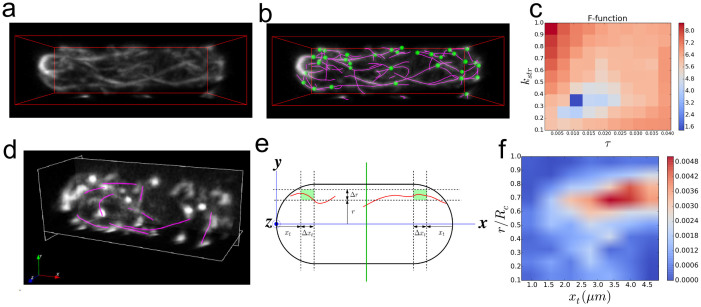
Analysis of actin cables in fission yeast cell imaged by confocal microscopy. (a) Volume rendered image of actin cables labeled by GFP-CHD (11.3 × 4.1 × 5.2 μm). The cell was treated with CK-666 to inhibit actin patch formation. (b) Cell image with extracted SOACs (magenta) and junctions (green). (c) F-function (*t* = 2, *c* = 1.8) shows the optimal τ = 0.009, *k*_str_ = 0.3. (d) Volume rendered image of actin cables labeled by GFP-CHD in fission yeast cell not treated with CK-666 and extracted SOACs (magenta). The manual editing capabilities of SOAX allowed deletion of SOACs and SOAC segments going through actin patches. (e) Schematic of fission yeast sphero-cylindrical geometry and measurement of SOAC segment density as function of distance to closest cell tip, *x_t_*, and radial distance *r* from axis of cylindrical symmetry (Δ*r* and Δx_t_ are the corresponding increments). (f) SOAC point density distribution vs *x_t_* and *r*/*R*_c_, where *R*_c_ is cell radius, averaged for *n* = 30 cells. For distances longer than 2 μm from the cell tips, actin cables localize away from the cell middle and close to the outer cell membrane. Actin cables for *x_t_* < 1 μm cannot be detected reliably due to the high density of actin patches at the cell tips.

Another challenge in the analysis of cell images is inhomogeneous background and presence of features in the image that need to be excluded in the analysis. An example is the analysis of actin cable networks in yeast and plant cells (Fig. 6). Actin cables contribute to cell polarization and measurements of their properties in wild type and mutant cells can provide information on the basic biophysical mechanisms of cell growth^37^.

Fission and budding yeast contain however two types of actin structures in interphase: actin cables and actin patches, both of which are present in images where actin filaments are marked by GFP-CHD, an actin filament side-binding protein^37^. Eliminating the actin patches with the drug CK-666 (Fig. 6a) allows SOAX to segment the actin cable network in fission yeast (Fig. 6b), after using the F-function to select a good extraction result from a set of 14 optimal candidates out of 110 combinations of ridge threshold τ and stretch factor *k*_str_ (distributed in the range 0.001 < τ < 0.041 and 0.1 < *k*_str_ < 1, similar to Fig. 3) (Fig. 6c).

SOAX's functionality further allows users to extract actin cable networks even in the presence of actin patches (Fig. 6d). We provide two mechanisms that reduce the influence of bright actin patches on the extraction of the actin cable network. Considering that actin patches usually have much higher intensity values than actin cables, we first reduce the number of SOACs going through patches by limiting initialization and SOAC elongation to within a specified allowable intensity range. Second, we use the post-convergence manual editing functions in SOAX to delete the few SOACs that initialized on dim patches and to correct the SOACs that are attracted to the patches from neighboring actin cables. The post-convergence manual editing allows deletion of the few SOACs that initialized on dim patches. It also enables correcting the kinks and sharp bends due to actin patches near the body or tips of actin cables that act as basins of attraction for the SOACs. This is done by examining the centerline of the cables near actin patches, using the slicing planes in three directions. After identifying SOACs that do not represent the centerline of the cables, SOAX provides functionality to extend or delete the SOAC segment going through the patch and to specify a point linking two broken ends. The user can then let the SOAC evolve for a few iterations to remove kinks.

Applying SOAX to multiple cells allowed us to quantitatively measure the average spatial distribution of actin cables in wild type fission yeast cells. Since fission yeast has cylindrical symmetry, we measured the SOAC point density as function of distance to the cell tips and the cell's axis of cylindrical symmetry, which were found manually (Fig. 6e). We found that for distances longer than 2 μm from the cell tips, actin cables localize away from the cell middle and close to the outer cell membrane. This result suggests that the excluded volume of the nucleus in the cell middle and/or outward pulling forces from myosin V in the cortical ER^38^ (that localizes close to the outer membrane) play a role in actin cable positioning with the cell. Use of SOAX in future studies with fission yeast mutants will help resolve the underlying mechanism.

## Discussion

We showed how SOAX provides a powerful and user-friendly platform for extracting and quantifying biopolymer networks imaged by confocal microscopy in 3D. The accompanying parameter optimization program helps the user select the best extraction results among candidates for subsequent analysis. Once a good parameter set is found for one image, it can be re-used for a set of images obtained under the same conditions, with care to use consistent image offset background subtraction and rescaling (the latter can be adjusted in SOAX but unless otherwise specified the program rescales the image so that the maximum intensity is 1). The user can also manually edit the best result to further improve it. The SOAX software and its parameter optimization program are open source and available for download at http://www.cse.lehigh.edu/~idealab/soax/.

The results in Figures 3–6 show the application of SOAX to 3D images. However, SOAX can be equally well applied to 2D network images (Supplementary Fig. 2), which are also very common in both in vitro and live cell experiments. This works by treating a 2D image as a 3D image with constant *z* coordinate. The main difference is the computation of the magnitude of the SOAC tip stretching force, where the local background neighborhood turns from a 2D annulus to a pair of 1D line segments.

SOAX works best for images of filaments or bundles of filaments that are sufficiently dilute such that the PSF width is narrower than their average separation. Since SOAC image forces are computed by comparing the intensity along the SOAC to the local background, analysis of 3D wide-field microscopy images that include significant out-of-focus background light would require deconvolution. SOAX resamples the image before the extraction process to make the voxel size isotropic along all axis directions. Sampling along the z direction should be higher than the Nyquist rate, otherwise artefacts such as false positive SOACs aligning along the *z*-direction may arise. The anisotropic resolution of confocal microscopy along the *z* direction will impact SOAX results so care must be taken in the analysis to avoid systematic errors. SOAX specifically provides selective initialization and damped elongation along *z*-axis to mitigate this problem: users can choose to only initialize SOACs along *x* and *y* axis directions; a “damp *z*” option suppresses elongation for SOACs aligned along z.

The network extraction time for the single frame images presented in this work is of order minutes on a desktop computer, for optimal parameters. For these parameters, most of the computation time was spent evolving SOACs rather than checking overlap (sets of consecutive points along SOACs for which the distance is less than a defined threshold^23^, see Supplementary Note 1), so we were in the regime where our algorithm scales approximately linearly with image size. Pairwise overlap checking becomes limiting for much larger images.

SOAX will also be helpful for analyzing the temporal evolution of biopolymer networks. This can be achieved by batch processing all frames of a time lapse image sequence. The network structure in each frame from a 2D/3D time-lapse sequence can be extracted and analyzed individually. SOAX can load simultaneously extraction results of different time frames for visual comparison of changes in filament orientation, curvature and spatial distribution over times. By comparing changes of junction location and filament shape and distribution in time, one can thus quantify biopolymer network (de)polymerization dynamics as well as mechanical deformations.

## Methods

### Ridge threshold τ

We initialize SOACs on the centerlines of filaments by locating intensity ridge points^23^ (Supplementary Note 1). We define a ridge point in axis *k* to be the image location ***x*** where intensity is a local maximum along axis *k*. A ridge point is detected by searching for a sign change in the *k*^th^ component of gradient ∂*_k_I*(***x***) where *I*(***x***) is image intensity. The magnitude of the sign change must be larger than ridge threshold τ. To allow initialization along filaments of different width, there is no restriction on the distance between the positions of the positive and negative intensity gradients (Supplementary Eq. 6). We assume image intensity in the range 0 and 1 (intensity rescaling can be performed within SOAX). Units of τ and other parameters are in rescaled intensity units and pixels.

### Stretch factor *k*_str_

The external force exerted on a SOAC is a weighted combination of image force and stretching force, ***F****_ext_* = *k_img_****F****_img_* + *k_str_****F****_str_*. We use units in which *k*_img_ = 1 and vary *k*_str_ only. The image force is proportional to the magnitude of local image intensity gradient. The stretching force is applied to SOAC tips and is proportional to the local image contrast, 1 − *I_b_*/*I_f_*, where *I_f_* is intensity at SOAC tip and background *I_b_* is intensity calculated by sampling uniformly within concentric circles or ellipses from a plane perpendicular to the SOAC tangent vector. The reason we sample the background to the sides of the SOAC is to allow them to extend and form junctions with bright filaments that lie ahead of them^23^.

### SOAC evolution

SOACs evolve according to internal forces representing bending and stretching stiffness and external forces described above (see Supplementary Note 1).

### F-function

The F-function evaluates a result based on the SNR of the local neighborhood of a SOAC. The portion of resultant SOACs that lie in regions with local SNR below *t* are considered uncertain thus penalized from the total length of SOACs in proportion to parameter *c* (Supplementary Note 2 and Eq. 8). Unlike the Hausdorff distance and vertex error, the F-function does not require ground truth to evaluate the optimal τ and *k*_str, _therefore it can also be used for selecting optimal parameters in experimental images that do not have ground truth. However, the soundness of optimal parameters suggested by the F-function depends on the values of *t* and *c*. Using synthetic images of varying SNR we find that valid *t* and *c* can be found within a fixed small range (Supplementary Note 2). Thus, we vary *t* and *c* in a small range to generate a set of “optimal candidates” for the user to choose from. This procedure for guided fine-tuning is necessary, especially for low SNR images where a narrower range of *t* and *c* is allowed and these values are sensitive to the precise definition of the local SNR. The small range on the values of *t* and *c* we choose are found by synthetic experiments. We construct synthetic images with different SNRs due to either shot noise or Gaussian noise (for the latter see Supplementary Note 2). For the shot noise case (Fig. 2) we first assign the background intensity 30 and the foreground intensity 90 to the centerline pixels specified by a set of ground truth SOACs and then convolve it with an anisotropic Gaussian 3D kernel with σ = (1.73, 1.73, 5.0) pixels, which simulates the point spread function. Finally, we scale the image intensity by 0.4 and use the resulting pixel intensity *I*_p_ to apply a Poisson distributed random variable to each voxel, with average *I*_p_.

### SOAC point density and intensity calculation

For the emulsion droplet in Figure 3 we used the following procedure based on the extracted SOAC points. Given that the droplet is a sphere of *R* pixels centered at ***p*_0_**, to calculate the SOAC point density, we counted the number of SOAC points ***p*** with with *r* < ||***p*** − ***p***_0_|| ≤ *r* + 1, for all 0 ≤ *r* ≤ *R* − 1 (here *r* is in pixels); this number was then divided by the surface area 4*πr*^2^. For the SOAC point intensity we measured the average intensity at the same points ***p***. The image intensity versus *r* is computed in a similar way by averaging the intensity of voxels with distance to the center in the interval (*r*, *r* + 1].

### Filament orientation in 3D

Filament orientation in Figures 4 and 5e was quantified by azimuthal angle *φ* and polar angle *θ* using a spherical coordinate system. We compute these angles for each SOAC segment denoted by a vector ***a*** = [*x, y, z*], which is the line segment between consecutive SOAC points. Since we cannot distinguish the polarity of the filament, we consider the orientation of +***a*** and −***a*** to be the same; thus we define the range of *φ* and *θ* to be (−90°, 90°] and [0, 180°), respectively, both with a period of π. To calculate the angles we use *φ* = atan(*y*/*x*) and *θ* = acos(z/||***a***||) for positive *x*. When *x* < 0, we first invert ***a*** before applying these equations. When ***a*** is aligned along the *z*-axis (*x* = *y* = 0) then *φ* = *θ* = 0. When ***a*** is on the *y*-*z* plane but not along *z*-axis (*x* = 0, *y* ≠ 0), then we invert ***a*** when *y* < 0 and use *φ* = 90°, *θ* = acos(z/||a||). In Supplementary Figure 1 we also computed the distribution of the single radial angle *γ* (0° ≤ *γ* ≤ 90°), which is the angle between ***a*** and the outward radial direction from a pre-defined center.

### Filament curvature

The curvature κ in Figure 5f is defined as the magnitude of the rate of change of unit tangent vector ***t***(*s*) with respect to arc length *s*, κ = ||d**t**/ds||. We estimate unit tangent vectors using SOAC points Δ_c_ = 8 pixels apart in arc length, a distance which is large enough to represent the curvature of the filament in the image and is independent of the intrinsic stiffness of the SOAC^33^. Specifically, *κ*(*s*) = ||***t***(*s* + Δ*_c_*/2) − ***t***(*s* − Δ*_c_*/2)||/Δ*_c_*, where 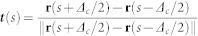. To avoid measuring curvature at junction points, we cut the SOACs at all junctions before calculating the curvature distribution.

### Actin bundles in emulsion droplets and confocal microscopy

Actin was polymerized in the presence of 1.2 μM fascin and 0.95 μM streptavidin in an actin polymerization buffer (25 mM imidazole-HCl (pH 7.4), 50 mM KCl, 2 mM MgCl_2_, 1 mM DTT, 0.1 mM MgATP, 1.33 mg/mL creatine phosphate, 2.48 mg/mL creatine phosphokinase, 0.1 mg/mL glucose oxidase, 0.1 mg/mL catalase and 280 mM sucrose). The actin concentration was 14 μM, including 34 mole% of AlexaFluor 488-actin and 0.42 mole% of biotinylated actin. An oil-lipid mixture was prepared by dissolving a lipid mixture of DOPC, 40 mole% DOPS and 1 mole% biotinylated lipid (Biotin-x-DHPE) in mineral oil containing 2% (w/w) Span 80 at a total lipid concentration of 0.5 mg/mL. Water-in-oil droplets were prepared using a flow-focusing microfluidic device, obtaining a narrow size distribution, as described elsewhere^39^,^40^. Droplets were observed at room temperature by an inverted microscope (DMIRB, Leica) equipped with a confocal spinning disc scan head (Yokogawa), a EM-CCD camera (C9100, Hamamatsu Photonics) and a 100x oil immersion objective. The intensity analysis in Figure 3k shows uneven actin encapsulation among droplets.

### Hela cells and confocal microscopy

We used HeLa cells stably expressing GFP-tubulin^41^. The culture medium was prepared with MEM medium; Glutamine (200 mM, 6 ml/500 ml media); sodium pyruvate (100 mM, 1 ml/100 ml media); fetal bovine serum (10%); penicillin-streptomycin antibiotic (100X, 1 ml/100 ml media). Cells were cultured in a flask in a NuAire CO_2_ incubator (5% CO_2_ at 37°C). We used an Olympus FV1000 confocal microscope with an Olympus UPLAN 100X oil immerse objective (NA = 1.3). Image resolution in x-y plane was 62 nm/pixel and z-step size 100 nm.

### Fission yeast methods and confocal microscopy

The strain FC1218 (*h^−^ 41nmt1-GFP-CHD (rng2)-leu1^+^ ade6-M216 leu1-32 ura4-D18*^42^) was cultured at 25°C in the rich media YE5S for 24 h and then washed into the minimal medium without thiamine EMM5S for 24 h at log phase to induce the expression of GFP-CHD. To reduce the interference from actin patches, cells were treated with 100 μM Arp2/3 complex inhibitor CK-666 from a 10-mM stock in DMSO at 25°C for 10 min before imaging. Images were collected using an UltraVIEW ERS spinning-disk confocal microscope (Perkin Elmer, Waltham, MA) with a 100x/1.4 NA Plan-Apo objective lens (Nikon, Melville, NY) as described before^43^. An ORCA-AG CCD camera (Hamamatsu, Bridgewater, NJ) was used without binning. Z stacks spanning 5 μm with a 0.2 μm spacing were collected.

## Author Contributions

T.X., D.V. and X.H. designed algorithms. T.X. developed code and analyzed data with input from other authors. E.Y. tested software and analyzed data. F.C.T. and G.K. performed experiments with emulsion droplets. W.N. performed experiments with HeLa cells. I.J.L. and J.Q.W. performed experiments with yeast cells. All authors contributed to writing the paper.

## Supplementary Material

Supplementary Information Supplementary Information

Supplementary Information Movie 1

Supplementary Information Movie 2

## References

[b1] Köhler S., Schaller V. & Bausch A. R. Structure formation in active networks. Nat. Mater. 10, 462–8 (2011). 21516093 10.1038/nmat3009

[b2] Reymann A.-C. *et al.* Actin network architecture can determine myosin motor activity. Science 336, 1310–4 (2012). 22679097 10.1126/science.1221708 PMC3649007

[b3] Sanchez T., Chen D. T. N., DeCamp S. J., Heymann M. & Dogic Z. Spontaneous motion in hierarchically assembled active matter. Nature 491, 431–4 (2012). 23135402 10.1038/nature11591 PMC3499644

[b4] Bailey M., Conway L., Gramlich M. W., Hawkins T. L. & Ross J. L. Modern methods to interrogate microtubule dynamics. Integr. Biol. (Camb). 5, 1324–33 (2013). 24061278 10.1039/c3ib40124c

[b5] Piechocka I. K., Bacabac R. G., Potters M., Mackintosh F. C. & Koenderink G. H. Structural hierarchy governs fibrin gel mechanics. Biophys. J. 98, 2281–9 (2010). 20483337 10.1016/j.bpj.2010.01.040 PMC2872216

[b6] Kim E. *et al.* Correlation between fibrin network structure and mechanical properties: an experimental and computational analysis. Soft Matter 7, 4983 (2011).

[b7] Cardona A. & Tomancak P. Current challenges in open-source bioimage informatics. Nat. Methods 9, 661–5 (2012). 22743770 10.1038/nmeth.2082

[b8] Beil M., Braxmeier H., Fleischer F., Schmidt V. & Walther P. Quantitative analysis of keratin filament networks in scanning electron microscopy images of cancer cells. J. Microsc. 220, 84–95 (2005). 16313488 10.1111/j.1365-2818.2005.01505.x

[b9] Stein A. M., Vader D. A., Jawerth L. M., Weitz D. A. & Sander L. M. An algorithm for extracting the network geometry of three-dimensional collagen gels. J. Microsc. 232, 463–75 (2008). 19094023 10.1111/j.1365-2818.2008.02141.x

[b10] Lück S., Sailer M., Schmidt V. & Walther P. Three-dimensional analysis of intermediate filament networks using SEM tomography. J. Microsc. 239, 1–16 (2010). 20579265 10.1111/j.1365-2818.2009.03348.x

[b11] Weichsel J., Urban E., Small J. V. & Schwarz U. S. Reconstructing the orientation distribution of actin filaments in the lamellipodium of migrating keratocytes from electron microscopy tomography data. Cytometry 81, 496–507 (2012). 22499256 10.1002/cyto.a.22050

[b12] Herberich G., Ivanescu A., Gamper I., Sechi A. & Aach T. Analysis of length and orientation of microtubules in wide-field fluorescence microscopy. Pattern Recognit. 6376, 182–191 (2010).

[b13] Basu S., Dahl K. N. & Rohde G. K. Localizing and extracting filament distributions from microscopy images. J. Microsc. 250, 57–67 (2013). 23458491 10.1111/jmi.12018 PMC3638952

[b14] Winkler C., Vinzenz M., Small J. V. & Schmeiser C. Actin filament tracking in electron tomograms of negatively stained lamellipodia using the localized radon transform. J. Struct. Biol. 178, 19–28 (2012). 22387240 10.1016/j.jsb.2012.02.011

[b15] Rigort A. *et al.* Automated segmentation of electron tomograms for a quantitative description of actin filament networks. J. Struct. Biol. 177, 135–44 (2012). 21907807 10.1016/j.jsb.2011.08.012

[b16] Krauss P., Metzner C., Lange J., Lang N. & Fabry B. Parameter-free binarization and skeletonization of fiber networks from confocal image stacks. PLoS One 7, e36575 (2012). 22606273 10.1371/journal.pone.0036575 PMC3351466

[b17] Meijering E. *et al.* Design and validation of a tool for neurite tracing and analysis in fluorescence microscopy images. Cytometry. A 58, 167–76 (2004). 15057970 10.1002/cyto.a.20022

[b18] Pool M., Thiemann J., Bar-Or A. & Fournier A. E. NeuriteTracer: a novel ImageJ plugin for automated quantification of neurite outgrowth. J. Neurosci. Methods 168, 134–9 (2008). 17936365 10.1016/j.jneumeth.2007.08.029

[b19] Peng H., Ruan Z., Long F., Simpson J. H. & Myers E. W. V3D enables real-time 3D visualization and quantitative analysis of large-scale biological image data sets. Nat. Biotechnol. 28, 348–53 (2010). 20231818 10.1038/nbt.1612 PMC2857929

[b20] Dehmelt L., Poplawski G., Hwang E. & Halpain S. NeuriteQuant: an open source toolkit for high content screens of neuronal morphogenesis. BMC Neurosci. 12, 100 (2011). 21989414 10.1186/1471-2202-12-100 PMC3208608

[b21] Yuan X., Trachtenberg J. T., Potter S. M. & Roysam B. MDL constrained 3-D grayscale skeletonization algorithm for automated extraction of dendrites and spines from fluorescence confocal images. Neuroinformatics 7, 213–32 (2009). 20012509 10.1007/s12021-009-9057-y PMC2844542

[b22] Wang Y., Narayanaswamy A., Tsai C.-L. & Roysam B. A broadly applicable 3-D neuron tracing method based on open-curve snake. Neuroinformatics 9, 193–217 (2011). 21399937 10.1007/s12021-011-9110-5

[b23] Xu T., Vavylonis D. & Huang X. 3D actin network centerline extraction with multiple active contours. Med. Image Anal. 18, 272–84 (2014). 24316442 10.1016/j.media.2013.10.015 PMC3945675

[b24] Unnikrishnan R., Pantofaru C. & Hebert M. Toward objective evaluation of image segmentation algorithms. IEEE Trans. Pattern Anal. Mach. Intell. 29, 929–44 (2007). 17431294 10.1109/TPAMI.2007.1046

[b25] Mayerich D., Bjornsson C., Taylor J. & Roysam B. NetMets: software for quantifying and visualizing errors in biological network segmentation. BMC Bioinformatics 13 Suppl 8S7 (2012). 22607549 10.1186/1471-2105-13-S8-S7 PMC3355337

[b26] Kohlberger T., Singh V., Alvino C., Bahlmann C. & Grady L. Evaluating segmentation error without ground truth. Med. Image Comput. Comput. Assist. Interv. 15, 528–36 (2012). 23285592 10.1007/978-3-642-33415-3_65

[b27] Zhang H., Fritts J. E. & Goldman S. A. Image segmentation evaluation: A survey of unsupervised methods. Comput. Vis. Image Underst. 110, 260–280 (2008).

[b28] Fawcett T. An introduction to ROC analysis. Pattern Recognit. Lett. 27, 861–874 (2006).

[b29] Hripcsak G. & Rothschild A. S. Agreement, the f-measure, and reliability in information retrieval. J. Am. Med. Inform. Assoc. 12, 296–8 (2005). 15684123 10.1197/jamia.M1733 PMC1090460

[b30] Alvarado J., Mulder B. M. & Koenderink G. H. Alignment of nematic and bundled semiflexible polymers in cell-sized confinement. Soft Matter 10, 2354–64 (2014). 24623093 10.1039/c3sm52421c

[b31] Fošnarič M., Iglič A., Kroll D. M. & May S. Monte Carlo simulations of a polymer confined within a fluid vesicle. Soft Matter 9, 3976 (2013).

[b32] Ostermeir K., Alim K. & Frey E. Buckling of stiff polymer rings in weak spherical confinement. Phys. Rev. E 81, 061802 (2010). 10.1103/PhysRevE.81.061802 20866431

[b33] Smith M. B. *et al.* Segmentation and tracking of cytoskeletal filaments using open active contours. Cytoskeleton 67, 693–705 (2010). 20814909 10.1002/cm.20481 PMC3020657

[b34] Brangwynne C. P., MacKintosh F. C. & Weitz D. A. Force fluctuations and polymerization dynamics of intracellular microtubules. Proc. Natl. Acad. Sci. U. S. A. 104, 16128–33 (2007). 17911265 10.1073/pnas.0703094104 PMC2042173

[b35] Bicek A. D., Tüzel E., Kroll D. M. & Odde D. J. Analysis of microtubule curvature. Methods Cell Biol. 83, 237–68 (2007). 17613311 10.1016/S0091-679X(07)83010-X

[b36] Brangwynne C., Koenderink G., MacKintosh F. & Weitz D. Nonequilibrium Microtubule Fluctuations in a Model Cytoskeleton. Phys. Rev. Lett. 100, 118104 (2008). 18517833 10.1103/PhysRevLett.100.118104

[b37] Drake T., Yusuf E. & Vavylonis D. A systems-biology approach to yeast actin cables. Adv. Exp. Med. Biol. 736, 325–35 (2012). 22161338 10.1007/978-1-4419-7210-1_19 PMC3241217

[b38] Zhang D., Vjestica A. & Oliferenko S. Plasma membrane tethering of the cortical ER necessitates its finely reticulated architecture. Curr. Biol. 22, 2048–52 (2012). 23041194 10.1016/j.cub.2012.08.047

[b39] Ménétrier-Deremble L. & Tabeling P. Droplet breakup in microfluidic junctions of arbitrary angles. Phys. Rev. E 74, 035303 (2006). 10.1103/PhysRevE.74.035303 17025697

[b40] Nishimura K., Suzuki H., Toyota T. & Yomo T. Size control of giant unilamellar vesicles prepared from inverted emulsion droplets. J Colloid Interface Sci. 376, 119–125 (2012). 22444482 10.1016/j.jcis.2012.02.029

[b41] Arakawa Y., Cordeiro J. V & Way, M. F11L-mediated inhibition of RhoA-mDia signaling stimulates microtubule dynamics during vaccinia virus infection. Cell Host Microbe 1, 213–26 (2007). 18005700 10.1016/j.chom.2007.04.007

[b42] Martin S. G. & Chang F. Dynamics of the formin for3p in actin cable assembly. Curr. Biol. 16, 1161–70 (2006). 16782006 10.1016/j.cub.2006.04.040

[b43] Laporte D., Coffman V. C., Lee I.-J. & Wu J.-Q. Assembly and architecture of precursor nodes during fission yeast cytokinesis. J. Cell Biol. 192, 1005–21 (2011). 21422229 10.1083/jcb.201008171 PMC3063137

