# Supplementary material for: SOAX: A software for quantification of 3D biopolymer networks
Source: Sci Rep. 2015 Mar 13;5:9081. doi: 10.1038/srep09081 (PMC4357869; doi:10.1038/srep09081)
Supplement: Supplementary Information [file srep09081-s1.pdf]

Supplementary Information for the Article: SOAX: A Software for Quantification of 3D Biopolymer Networks

Ting Xu, Dimitrios Vavylonis, Feng-Ching Tsai, Gijsje Koenderink, Wei Nie, Eddy Yusuf, I-Ju Lee, Jian-Qiu Wu, and Xiaolei Huang

Contents

1 Supplementary Note 1: Summary of the Multiple SOACs Method **2**

1.1 SOACs with Adaptive Stretching Force 2
1.2 Automatic Initialization of Multiple SOACs 4
1.3 Sequential Evolution of Multiple SOACs 4
1.4 Topology Configuration of the SOACs Network 5

2 Supplementary Note 2: Parameter Optimization using the F-function **7**

2.1 Definition of the F-function 7
2.2 Synthetic Images 8
2.3 Choice of Meta-parameters t, c of F-functions 10

List of Figures

1 Figure SN1: Estimation of local background around a SOAC tip. 3
2 Figure SN2: End overlap check. 5
3 Figure SN3: Topology configuration and junction location. 6
4 Figure SN4: Examples of synthetic images with Gaussian noise. 8
5 Figure SN5: Parameter optimization using F-function. 9
6 Figure SN6: Hausdorff distance of optimal extraction versus t, c. 10
7 Figure SN7: Vertex error of optimal extraction versus t, c. 11
8 Figure SN8: Hausdorff distance and vertex error averaged across noise levels. 12
1 Supplementary Figure 1: Radial Angle Distribution Analysis of Filaments in an Emulsion Droplet 13
2 Supplementary Figure 2: Example of 2D Network Extraction by SOAX . . . 14

1 Supplementary Note 1: Summary of the Multiple SOACs Method

In this section we review the multiple SOACs method [1] implemented in SOAX. The method involves three stages. First, multiple SOACs are initialized automatically on intensity ridges. Secondly, they evolve sequentially to extract the centerlines of a network without any overlap. Potential network junctions are also identified during their sequential evolution. In the last stage, network junctions are located and the network topology at junctions are configured so that SOACs go through them smoothly. The extraction result is a network of SOACs (each represented by a curve in 3D) linked by a set of junction points.

1.1 SOACs with Adaptive Stretching Force

SOACs are open-ended parametric active contour models with stretching forces applied at their two tips [2]. The contour can elongate or shrink while conforming to desired image features such as edges or intensity ridges. Mathematically, a 3D SOAC is a curve $\boldsymbol{r}(s) = \{x_0(s), x_1(s), x_2(s)\}^T, s \in [0, L]$ parameterized by arc length s, with L being its total curve length. A SOAC evolves by minimizing its contour energy functional $\mathcal{E}(\boldsymbol{r}(s)) = \mathcal{E}_{int}(\boldsymbol{r}(s)) + \mathcal{E}_{ext}(\boldsymbol{r}(s))$. Minimizing the internal energy functional $\mathcal{E}_{int}$ maintains the continuity and smoothness of the curve; minimizing the external energy functional $\mathcal{E}_{ext}$ pushes the curve towards desired image features. $\mathcal{E}_{int}(\boldsymbol{r}(s))$ is defined as

$$\mathcal{E}_{int}(\boldsymbol{r}(s)) = \int_0^L \alpha \left|\boldsymbol{r}'(s)\right|^2 + \beta \left|\boldsymbol{r}''(s)\right|^2 \,\mathrm{d}s, \tag{1}$$

where α and β are weights for the tension and rigidity of the curve, respectively. The external energy functional $\mathcal{E}_{ext}(\boldsymbol{r}(s))$ is a weighted sum of an image potential energy function $E_{img}(\boldsymbol{x})$ and a stretching energy $E_{str}(\boldsymbol{r}(s))$,

$$\mathcal{E}_{ext}(\boldsymbol{r}(s)) = \int_0^L k_{img} E_{img}(\boldsymbol{r}(s)) + k_{str} E_{str}(\boldsymbol{r}(s)) \,\mathrm{d}s, \tag{2}$$

where k_{img} and k_{str} are weights to control the strength of the image and stretching forces, respectively. The image potential energy field $E_{img}(\boldsymbol{x})$ is the convolution between an input image $I(\boldsymbol{x})$ and a Gaussian kernel with standard deviation σ: $E_{img}(\boldsymbol{x}) = I(\boldsymbol{x}) * G_\sigma(\boldsymbol{x})$. Thus the image force exerted on a SOAC can be derived as:

$$\nabla E_{img}(\boldsymbol{r}(s)) = \nabla(I * G_\sigma)(\boldsymbol{r}(s)) = (I * \nabla G_\sigma)(\boldsymbol{r}(s)). \tag{3}$$

The resulting forces point towards the center of bright intensity ridges (blue arrows in Figure 1b).

The stretching energy leads to the tangential force $\boldsymbol{F}(\boldsymbol{r}(s))$ exerted at a SOAC's tip,

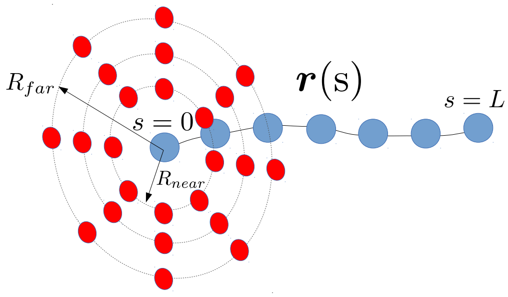

Figure SN 1: Estimation of local background around a SOAC tip. Red points are local background samples, all located on the plane perpendicular to the tangent at the SOAC tip ($\boldsymbol{r}(0)$ here) and delimited by radii R_{near} and R_{far}. A good choice for R_{near} is twice the point spread function width and a good choice for R_{far} is $2R_{near}$. The angle step size is $\pi/4$.

which makes the SOAC elongate or shrink:

$$\boldsymbol{F}(\boldsymbol{r}(s)) = \begin{cases} -F(\boldsymbol{r}(s))\frac{\boldsymbol{r}'(s)}{|\boldsymbol{r}'(s)|}, & s = 0 \\ F(\boldsymbol{r}(s))\frac{\boldsymbol{r}'(s)}{|\boldsymbol{r}'(s)|}, & s = L \\ 0, & 0 < s < L \end{cases} \tag{4}$$

where $F(\boldsymbol{r}(s))$ is the magnitude of stretching force. It is defined to be proportional to the local image contrast around the tip:

$$F(\boldsymbol{r}(s)) = \frac{I(\boldsymbol{r}(s)) - I_b(\boldsymbol{r}(s))}{I(\boldsymbol{r}(s))} = 1 - \frac{I_b(\boldsymbol{r}(s))}{I(\boldsymbol{r}(s))}, \quad s = 0, L \tag{5}$$

where $I(\boldsymbol{r}(s)|_{s=0,L})$ is the image intensity at a SOAC tip and $I_b(\boldsymbol{r}(s)|_{s=0,L})$ is the local background intensity around it. This definition of $F(\boldsymbol{r}(s))$ can remedy under-segmentation and over-segmentation in the presence of foreground and background intensity variations. We found that an effective way to estimate I_b is to sample local intensities around a tip on a set of perpendicular concentric circles of radii between R_{near} and R_{far} and then compute the average of these intensity samples (Fig. SN1).

To account for the anisotropic spreading along z that is typical in fluorescence images, the z coordinate of the local samples can be scaled by a fixed value (by default equal to 2.88). As a result, the local sampling region can be a concentric circle or an ellipse depending on the orientation of the tangent at a SOAC's tip.

The SOAC energy functional $\mathcal{E}(\boldsymbol{r}(s))$ is minimized by solving three independent Euler-Lagrange equations (one for each dimension). We use an iterative method [3] and each SOAC is resampled after each time step to keep the average separation between SOAC points at a desirable average distance. Thus a SOAC elongates in the presence of an extensile tip force and shrinks when there is zero or negative force.

1.2 Automatic Initialization of Multiple SOACs

We initialize SOACs on the centerlines of filaments by locating 3D intensity ridge points. We define a ridge point in axis k to be the image location of which intensity is locally maximum along axis $k, k = 0, 1, 2$. Thus a ridge point $\boldsymbol{x}$ can be detected by searching for the plus-to-minus sign change in the spatial derivatives of the smoothed image $\widetilde{I}(\boldsymbol{x})$, obtained by convolving the input image $I(\boldsymbol{x})$ with a Gaussian kernel of standard deviation σ. Let $\partial_k \widetilde{I}(\boldsymbol{x}) = \partial(G_\sigma(\boldsymbol{x}) * I(\boldsymbol{x}))/\partial x_k$ denote the image derivative along the kth axis direction. $\boldsymbol{x}$ is a ridge point in that direction if

$$\exists m > 0 : \begin{cases} \partial_k \widetilde{I}(\ldots, x_k - \lfloor m/2 \rfloor, \ldots) > \tau \\ \partial_k \widetilde{I}(\ldots, x_k + \lceil m/2 \rceil, \ldots) < -\tau \\ |\partial_k \widetilde{I}(\ldots, x_k + l, \ldots)| < \tau, \forall l \in (-\lfloor m/2 \rfloor, \lceil m/2 \rceil) \end{cases} \tag{6}$$

where ridge threshold $\tau > 0$ is the minimum change of intensity to trigger the detection of a ridge point. Here l, m are integers, and $m > 0$ is the width of the ridge.

We then identify candidate SOAC points as ridge points in at least two axis directions. Specifically, we define a SOAC candidate point along x axis as a point that is a ridge point in both of the other two axis directions, namely, y and z. Similarly, a SOAC candidate point along y is a ridge point in both x and z, and a SOAC candidate point along z is a ridge point in both x and y directions. Next, candidate SOAC points are linked to form initial SOACs.

Without assuming the orientation of filaments, we initialize SOACs separately along each axis direction: locally connected candidate points along x are linked in the ascending order of x coordinate to form an initial SOAC along x, and the process is repeated to form SOACs along y and z. The constructed initial SOACs may be redundant but they merge during their sequential evolution.

1.3 Sequential Evolution of Multiple SOACs

Initialized SOACs evolve until convergence, one after another, in a sequential manner. We introduce schemes to keep the converged network of SOACs free of overlap.

Detecting the presence of overlap for an evolving SOAC involves calculating the Euclidean distance from each point of the SOAC to each point of all prior converged SOACs. Overlaps of the evolving SOAC are all sets of consecutive points of which the distance to that of a converged SOAC is less than a predefined threshold d_e. Detecting overlap after each iteration is expensive and can dramatically slow down the SOAC evolution. If otherwise overlap is detected after the convergence of a SOAC, then computation time may be wasted on extracting filaments that have been extracted by other converged SOACs.

Here we adopt a balanced strategy. We divide SOAC overlaps into *end overlaps* and *body overlaps*. The former are the overlaps that start from the tip of an evolving SOAC. Detecting end overlap can be done effciently because we only need to check starting from two tips only, and stop at the first non-overlap point. Because of the lower computational cost, we check end overlap after each iteration. If an end overlap is found, we record the converged

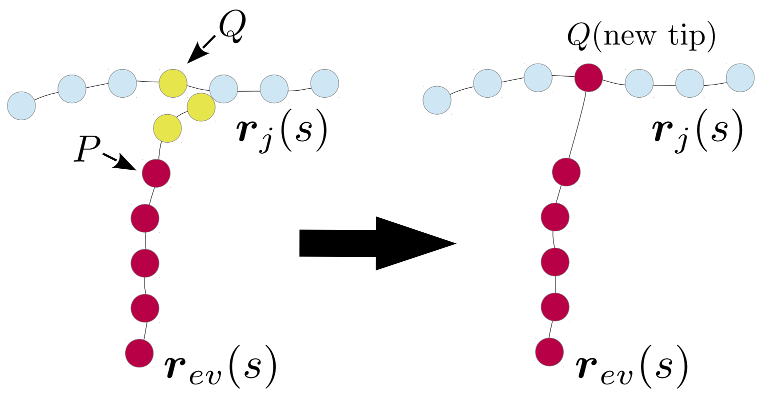

Figure SN 2: End overlap check after each SOAC iteration. (Left) The tip overlap (yellow) of an evolving SOAC $\boldsymbol{r}_{ev}$ with a converged SOAC $\boldsymbol{r}_j$. P is the first non-overlap point on $\boldsymbol{r}_{ev}$. Q is the point of $\boldsymbol{r}_j$ that is the closest to P. (Right) Deletion of the overlapping part, and estanlishment of Q as the new tip for $\boldsymbol{r}_{ev}$. Q is recorded as a "T-junction". After T-junction formation, $\boldsymbol{r}_{ev}(s)$ is resampled.

SOAC point that is closest to the first non-overlap point as a "T-junction" (Fig. SN2). The T-junction is used in the subsequent topology configuration stage.

The body overlap is an overlap that in the interior of an evolving SOAC and is usually caused by the SOAC body drifting towards other extracted filaments. Since body overlap check is typically computationally more expensive than end overlap (since we need to check each point of an evolving SOAC), it is performed once after each SOAC converges. The identified non-overlap parts become new independent SOACs that evolve again.

To detect loopy structures in a network and avoid infinite elongation of SOACs along them, we also check for self-intersection after each iteration. Specifically, we check if there are two SOAC points $\boldsymbol{r}_{ev}(s_1)$ and $\boldsymbol{r}_{ev}(s_2)$ that are sufficiently apart along the curve length but are spatially very close, i.e. $\|\boldsymbol{r}_{ev}(s_1) - \boldsymbol{r}_{ev}(s_2)\|_2 < d_e$ and $|s_1 - s_2| > \kappa$ where κ is a predefined threshold typically set to one tenth of the curve length L_{ev}. In the case of self-intersection, we divide $\boldsymbol{r}_{ev}$ into 3 SOACs which correspond to parts of $\boldsymbol{r}_{ev}(s)$ with $s \in [0, s_1], s \in [s_1, s_2], s \in [s_2, L_{ev}]$, respectively. The second SOAC will re-evolve as a closed curve active contour, followed by the other two SOACs.

1.4 Topology Configuration of the SOACs Network

The network of converged SOACs may not be topologically accurate because SOACs evolve sequentially (Fig. SN3(a)). Those SOACs that evolve first have a better chance to extend through filament intersections, along multiple filaments. When they elongate across intersections of filaments artificial corners may occur.

To retrieve a more accurate network topology, we use the fact that junctions in most cytoskeletal networks typically involve semi-flexible filaments crossing one another or side branches connected to straight filaments (such as those formed by the Arp2/3 complex along actin filaments). We use this smoothness constraint to compute a smooth branch-connection

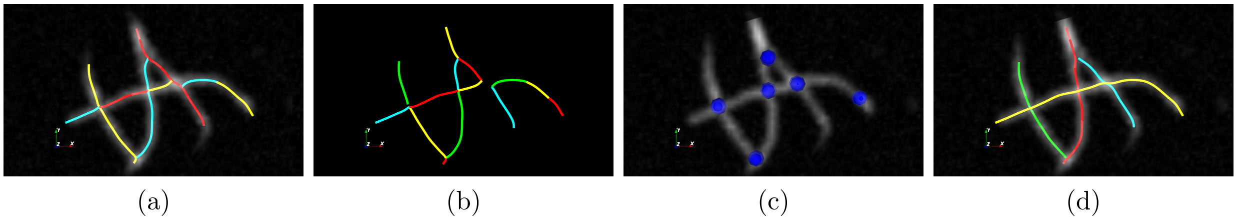

Figure SN 3: Topology configuration and junction location on a synthetic 3D image. (a) Converged network of SOACs (different colors). The SOAC connectivity does not correspond to a physically plausible topology. (b) Converged SOACs are dissected into segments (shown in different colors) at each T-junction. (c) Nearby T-junctions are clustered into a single higher-order network junction (blue spheres). Note the rightmost junction is not a false positive as there is a branch perpendicular to this image plane that is not distinguishable. (d) New SOACs formed by reconfiguration and linking of grouped segments.

configuration at junctions.

To retrieve a topologically correct network, we first cut converged SOACs at each recorded T-junction into "SOAC segments" (Fig. SN3(b)). SOAC segments of length less than d_g are discarded, where d_g is a predefined threshold. Next we construct an undirected graph $G = (V, E)$, where V is the set of end points of SOAC segments. For any two vertices, $u, v \in V$, we have an edge $(u, v) \in E$ if $\|u - v\|_2 < d_g$. We then we apply connected components analysis on G to detect clusters of vertices, where each cluster represents a network junction (Fig. SN3(c)).

Next we define the smoothness between a pair of end points as the angle between the tangent vectors of SOACs at the two end points. For each network junction, we greedily link up the smoothest pair of end points across it, until all pairs are linked or the angle between current smoothest pair is above than a threshold angle (the default threshold is $2\pi/3$). The SOAC segments that are linked up become one longer SOAC (Fig. SN3(d)). The linked SOACs are then evolved with their ends fixed for 100 iteration to remove kinks that resulted from prior evolution, cutting and linking. We finally prune the detected network junctions by deleting those that have only one SOAC crossing them.

References

[1] Xu, T., Vavylonis, D. & Huang, X. 3D actin network centerline extraction with multiple active contours. *Medical Image Analysis* **18**, 272–284 (2014).

[2] Li, H. *et al.* Automated actin filament segmentation, tracking and tip elongation measurements based on open active contour models. In *Biomedical Imaging: From Nano to Macro, IEEE International Symposium on*, 1302–1305 (2009).

[3] Kass, M., Witkin, A. & Terzopoulos, D. Snakes: Active contour models. *International Journal of Computer Vision* **1**, 321–331 (1988).

2 Supplementary Note 2: Parameter Optimization using the F-function

Two key parameters of the multiple SOACs method are the ridge threshold τ (Eq. 6) and stretch factor k_{str} (Eq. 2). As we use SOAX to quantify different types of biopolymer network images, τ, k_{str} and other parameters need to be adapted to obtain a good extraction. Our goal is to find optimal parameters from a predefined set without knowledge of ground truth.

2.1 Definition of the F-function

Our observation is that we can better trust extraction results on image regions with high local SNR as compared to those on regions with low SNR. So we propose a criterion based on a defined "F-function" that is a measure of an extraction result. When we have a set of SOACs obtained from a predefined set of τ and k_{str}, we can optimize the F-function on this set to obtain the optimal τ and k_{str}.

Given an extraction result consisting of N SOACs, the F-function is a function of τ, k_{str} and two meta-parameters t and c:

$$
\begin{aligned}
F(\tau, k_{str}, t, c) &= -L_{total} + cL_{<t} \\
&= -\sum_{i=1}^{N} L_i + c\sum_{i=1}^{N} \int_0^{L_i} [1 - H(f_{snr}(\boldsymbol{r}_i(s)) - t)]\,\mathrm{d}s \\
&= \sum_{i=1}^{N} \{(c-1)L_i - c\int_0^{L_i} H(f_{snr}(\boldsymbol{r}_i(s)) - t)\,\mathrm{d}s\}. \qquad (7)
\end{aligned}
$$

Here L_{total} measures the total length of all SOACs and $L_{<t}$ is the length of SOACs in image regions with local SNR below the threshold t, with $c > 1$ being the factor controlling how much low-SNR SOACs are penalized. In Equation 7, L_i is the length of the ith SOAC, $H(\cdot)$ is the Heaviside step function and $f_{snr}(\cdot)$ is a function that computes the local image SNR at a SOAC point. It is defined by

$$
f_{snr}(\boldsymbol{r}(s)) = \frac{I(\boldsymbol{r}(s)) - \mu_{lb}(\boldsymbol{r}(s))}{\sigma_{lb}(\boldsymbol{r}(s))}, \qquad (8)
$$

where $I(\boldsymbol{r}(s))$ is the interpolated image intensity at SOAC point $\boldsymbol{r}(s)$. Here μ_{lb} and σ_{lb} are the mean and standard deviation of local background intensities, respectively. The local background is sampled similarly as the computation of adaptive stretching force (Eq. 5) from perpendicular concentric circles of radii R_{near} and R_{far} around $\boldsymbol{r}(s)$. The only difference is that the local SNR is evaluated at every SOAC point rather than just SOAC tips.

Minimizing the F-function favors extraction results that are as complete as possible (large L_{total}) but penalizes the portions with low local SNR ($L_{<t}$). When t and c are chosen properly, the F-function can have a similar dependence on τ and k_{str} as the Hausdorff distance and vertex error (Fig. 2(b-c)) and similar optimal and region representing good extraction results

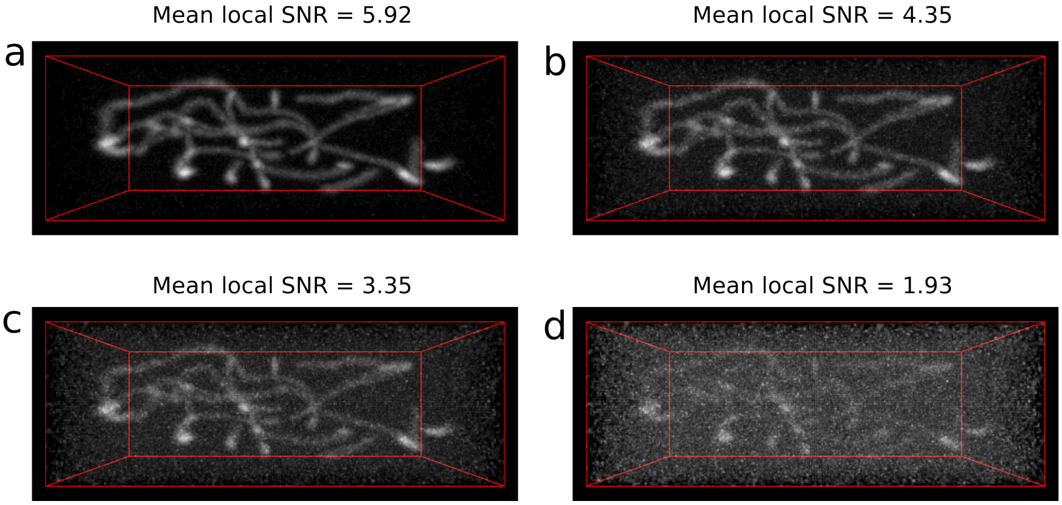

Figure SN 4: Examples of synthetic images with added Gaussian noise (different σ_n for each image). Images are constructed using known ground truth SOACs and convolved by an anisotropic Gaussian function to simulate the point spread function. (a) $\sigma_n = 1$; local SNR is $0.95 \leq f_{snr} \leq 14.72$ with a mean of 5.92. (b) $\sigma_n = 2$; local SNR is $0.17 \leq f_{snr} \leq 12.59$ with a mean of 4.35. (c) $\sigma_n = 3$; local SNR is $0 \leq f_{snr} \leq 9.81$ with a mean of 3.35. (d) $\sigma_n = 6$; local SNR is $0 \leq f_{snr} \leq 7.11$ with a mean of 1.93.

as the other two metrics (Fig. 2(d)). The optimal extraction result corresponds to the image very well (Fig. 2(e)) and is very close to the ground truth (Fig. 2(f)).

2.2 Synthetic Images

To evaluate our proposed optimization methods, we constructed synthetic images using the centerlines of a manually extracted actin cable network. We used various amounts of shot noise (Fig. 2) and Gaussian noise (Fig. SN4). For the shot noise case, see Online Methods. For the Gaussian noise images, we first assign the foreground intensity 20 to the centerline pixels specified by a set of ground truth SOACs and then convolve it with an anisotropic Gaussian kernel with $\sigma_{psf} = \{1.73, 1.73, 5.0\}$ pixels, which simulates the point spread function. The foreground intensity is scaled back to 20 after the convolution to generate a clean image. Finally, Gaussian noise with mean $\mu_n = 200$ and standard deviation $\sigma_n = \{0, 1, 2, \ldots, 7\}$ is added.

The mean local SNR of a synthetic image is calculated by evaluating f_{snr} (Eq. 8) at each point of the ground truth centerlines. In the following we use $R_{near} = 4$ and $R_{far} = 8$ pixels in the computation of adaptive stretching force (Eq. 5) and F-function (Eq. 7).

SOAX and the F-function achieve similar results on images with either shot or Gaussian noise. In Figure 2 of the main text we show one example of F-function minimization on a synthetic image with shot noise. Figure SN5 shows an additional example of finding the optimal τ and k_{str} by minimizing an F-function on an image with Gaussian noise and appropriate values of t, c.

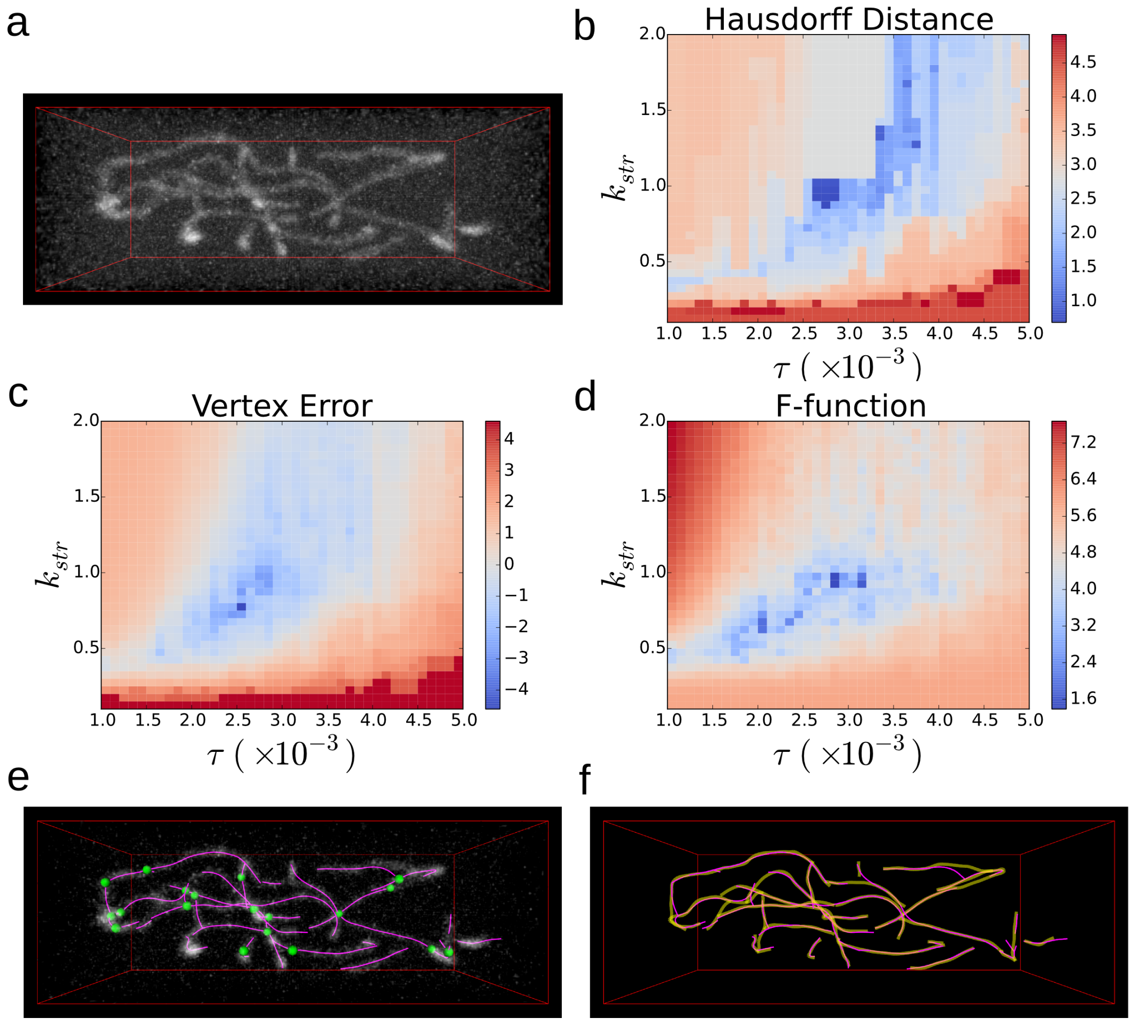

Figure SN 5: Parameter optimization on a synthetic image with Gaussian noise using the F-function. (a) Synthetic test image with mean local SNR = 3.35. (b-c) Hausdorff distance and vertex error (log-scale plots: $\ln(x - x_{min} + \epsilon)$, where x is the data value; x_{min} is the minimum value of all data; ϵ is a constant offset) computed between ground truth and results extracted using various values of the ridge threshold τ (normalized intensity change per pixel) and stretch factor k_{str} (pixels per time step). (d) The proposed F-function (log-scale) computed using $t = 3.9$ and $c = 1.2$. The optimal parameters are $\tau^* = 0.0028, k^*_{str} = 0.9$, selected by the minimum in F-function value. The optimal result has Hausdorff distance 8.71 pixels and vertex error 1.15 pixels compared to ground truth. (e) Optimal extraction result overlaid with original image in (a). (f) Optimal extraction result (magenta) overlaid with ground truth (translucent yellow).

2.3 Choice of Meta-parameters t, c of F-functions

For a given setting for the t, c meta-parameters, the optimal (τ^*, k^*_{str}) are those that minimize the F-function:

$$\{\tau^*, k^*_{str}\} = \underset{\tau \in \mathcal{T}, k_{str} \in \mathcal{K}}{\operatorname{argmin}} F(\tau, k_{str}, t, c), \tag{9}$$

where $\mathcal{T}$ and $\mathcal{K}$ are the sets of pre-selected values of τ and k_{str}, respectively. Here we find the proper range of t and c through experiments on synthetic images with Gaussian noise. We explore how the optimal (τ^*, k^*_{str}) depend on the setting of t, c.

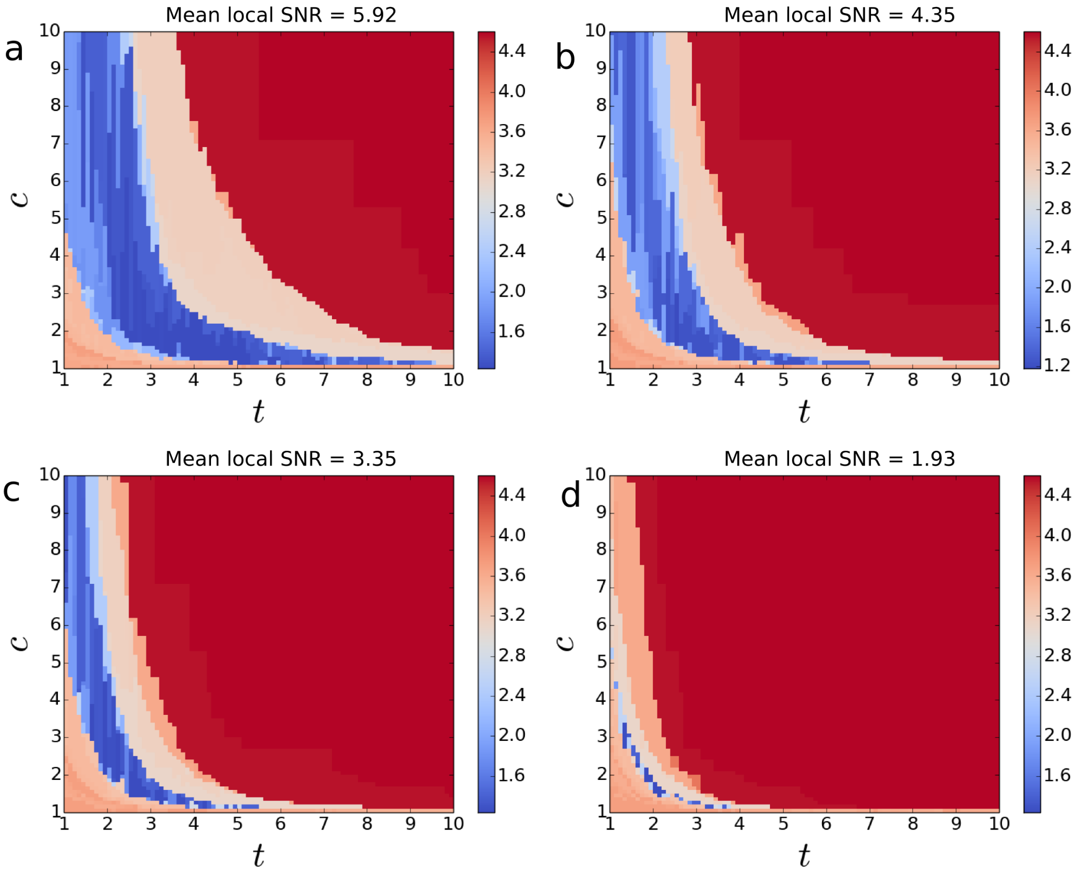

Figure SN 6: Log-scale Hausdorff distance of optimal extraction results compared to ground truth (log-scale: $\ln(x - x_{min} + \epsilon)$, where x is the data value; x_{min} is the minimum value of all data; ϵ is a constant offset). Each panel corresponds to one image in Figure SN4. Each optimal result minimizes the F-function defined by the corresponding t and c values. We use the same set of 2000 combinations of τ and k_{str} for each one of synthetic images. This figure shows that the range of good combination of (t, c) (dark blue color) shrinks as the image noise level increases.

We extracted network from synthetic images using various combination of τ and k_{str}. Here we choose $\mathcal{T} = \{0.002n | 1 \leq n \leq 50, n \in \mathbb{N}\}$ and $\mathcal{K} = \{0.05n | 1 \leq n \leq 20, n \in$

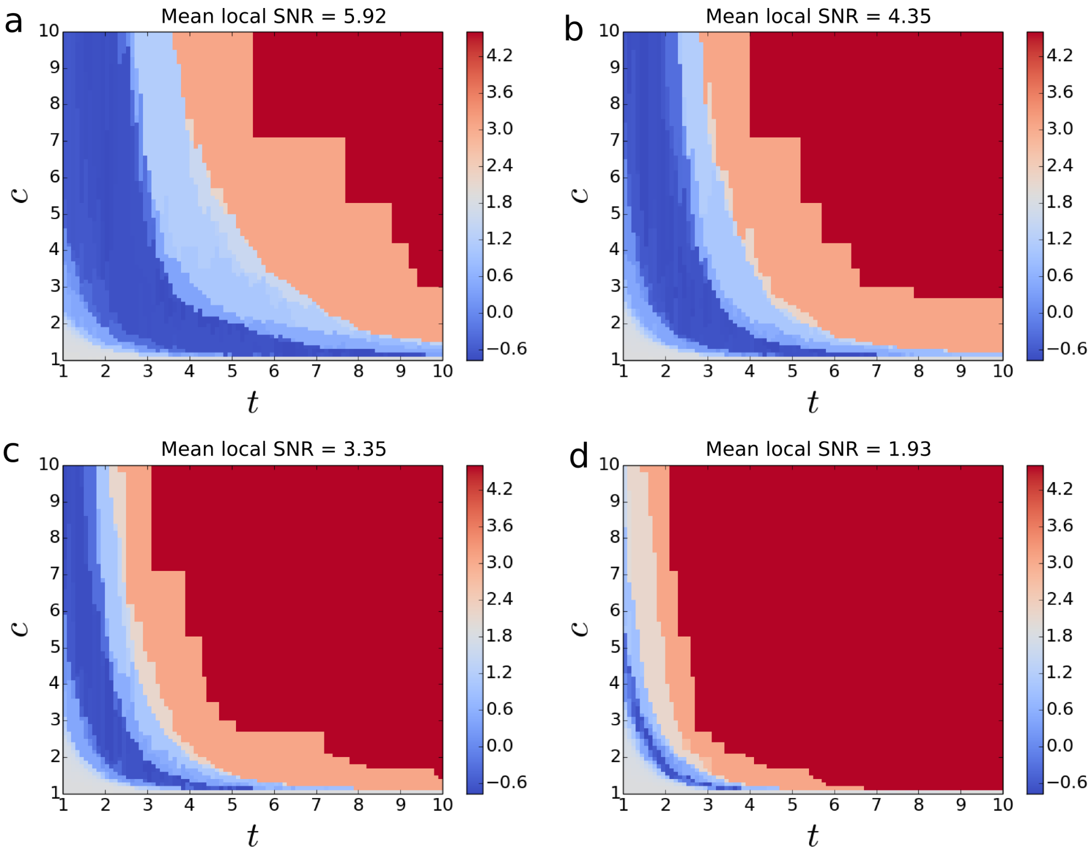

Figure SN 7: Same as Fig. SN 6 but for the vertex error of optimal extraction results versus t and c (log-scale: $\ln(x - x_{min} + \epsilon)$, where x is the data value; x_{min} is the minimum value of all data; ϵ is a constant offset). The vertex error also shows that the range of good combination of (t, c) (dark blue color) shrinks as the image noise level increases.

$\mathbb{N}\} \bigcup \{1 + 0.1n | 1 \leq n \leq 20, n \in \mathbb{N}\}$ to generate 2000 extraction results. We then vary t and c, both in the range $\{0.1n | 10 \leq n \leq 100, n \in \mathbb{N}\}$. For each pair of (t, c), an optimal extraction is found by minimizing the corresponding F-function. We then compare that result with the known ground truth using Hausdorff distance (Fig. SN6) and Vertex Error (Fig. SN7).

Figures SN6 and SN7 show that (1) t is inversely related to c in order to achieve good extraction results; (2) the number of (t, c) pairs that lead to good extraction decreases as the image SNR decreases, which means the optimal extraction is more sensitive to choice of (t, c) values when the image SNR is low. However we notice that a small range of (t, c) almost always lead to good extraction results. From the average Hausdorff distance and Vertex error across different levels of image noise (Fig. SN8), we can see it is adequate to choose $\{(t, c) | 1 < t < 5, 1 < c < 5, 3 < t + c < 6\}$ regardless of the input image SNR.

Based on the above results, we designed the following procedure to help users find optimal parameter values. A script provided in SOAX can be used to generate multiple extraction

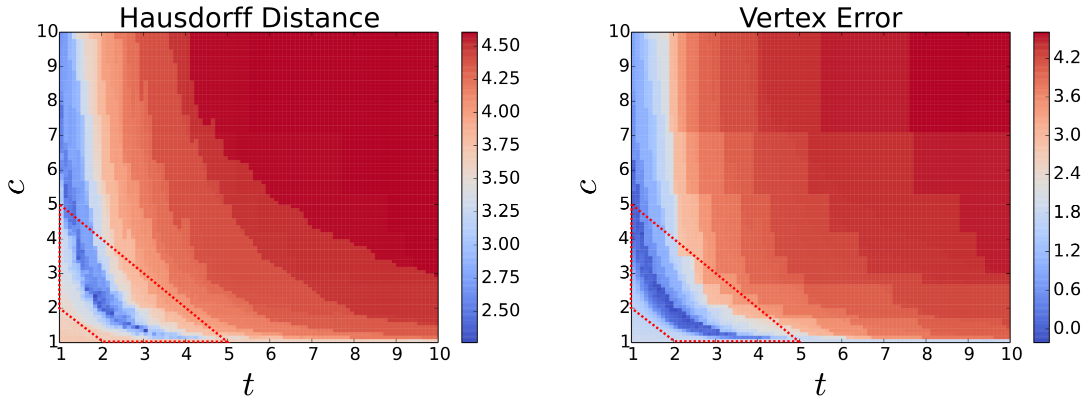

Figure SN 8: Log-scale Hausdorff distance and Vertex error against (t, c) averaged across 8 different image noise levels (including clean image). The Hausdorff distance and vertex error are computed against known ground truth. The trapezoidal region enclosed by dashed red lines shows the small but effective sampling region of (t, c) to generate candidate optimal results.

results with varying τ, k_{str} or other parameters. From this large set of results, a narrower set is selected by finding the optimal parameters using the F-function with a few t and c sampled within the above pre-defined small range. We have found that allowing the user to pick from this small set of candidate results works well with different types of experimental images that have varying SNR and different features (see main text). While some results in the set are not good, especially for very noisy images, the user can usually find a result that is good. Once a parameter set is found, the same parameters can be applied to additional images taken under the same conditions, or for a larger part of the image.

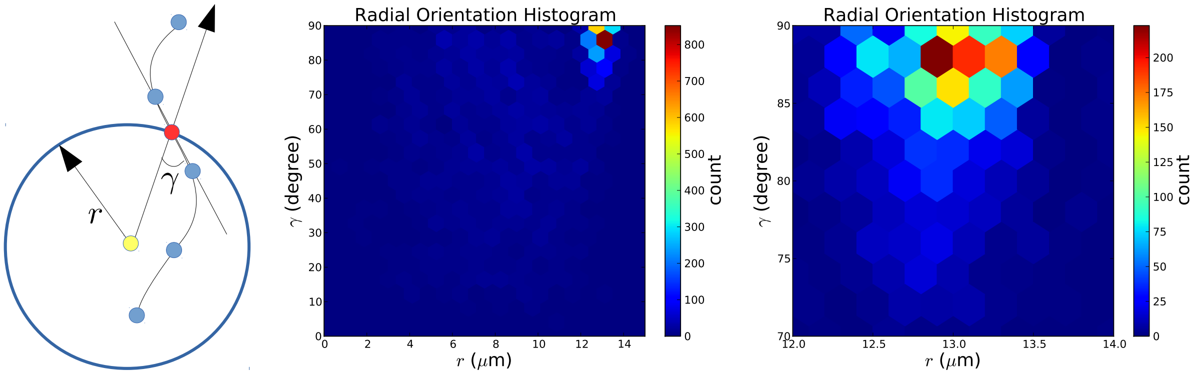

Supplementary Figure 1: An additional analysis method implemented in SOAX is analysis of the radial SOAC orientation distribution. Left: Definition of angle γ, which is the angle between the tangent of a SOAC segment and the radial direction. The red point is the mid point of two consecutive SOAC points. Middle: The distribution of γ as a function of radius r. This heat map shows that actin bundles encapsulated in an emulsion droplet align parallel to the boundary surface when they are near it. Right: A close up view of the top right part of the middle panel.

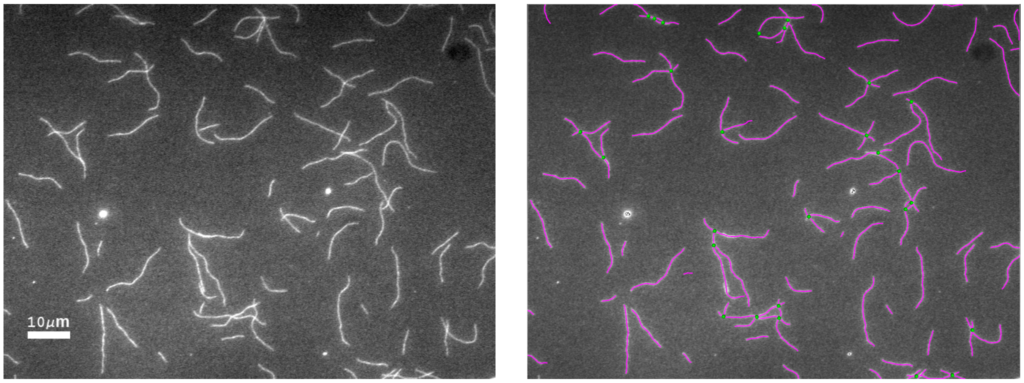

Supplementary Figure 2: An example of network extraction on a 2D Total Internal Reflection Fluorescence Microscopy (TIRFM) image of actin filaments growing on a glass slide (from Fujiwara et al., Proceedings of the National Academy of Sciences **104**, 8827-8832, 2007). Left: input image. Right: SOAX result overlaid with the input image showing the extracted contours and junction points.
